# A Case of Mortification of the Leg

**Published:** 1787

**Authors:** Joseph Brandish

**Affiliations:** Surgeon at Alcester, in Warwickshire


III. *A Cafe of Mortification of the Leg, by Mr.* Jofeph Brandifh, *Surgeon at Alcefter, in Warwickfhire. Communicated in a Letter to Mr.* Henry Cline, *Surgeon of St. Thomas's Hofpital, and Reader of Anatomy in London; and by him to Dr.* Simmons.

THE treatment of mortification has undergone confiderable alteration, fince it has been been obferved that ftimulants, which were formerly thought neceffary, only ferve to exafperate the difeafe.

The practice of removing a mortified part, with an intention of preventing it from fpreading, has been long fince difcontinued, being rarely attended with fuccefs. But when the mortification has ceafed to extend, and a feparation has begun to take place between the found and difeafed part, the operation has frequently fucceeded. But whether amputation is in general neceffary at this time, may be doubted, as Nature is equal to the work of performing a complete feparation, and that in a few weeks, of which the following cafe is a remarkable inftance.

Thomas Warner, aged fifteen years, of Grafton, a village near Alcefter, in February, 1778, was pricked by a thorn, in the great toe of his left foot, which in four days became confiderably inflamed. A fluctuation being difcovered on the under part of the toe, an opening was made, and about a drachm of bloody matter difcharged. On the fixth day he was very feverifh, the foot was much inflamed, and the toe next the little one was livid, and had loft all all fenfation. In three weeks the mortification had extended to within four inches of the knee, where it ftopt; and, in ten days after, a complete feparation was effected, the tibia and fibula coming away entire, leaving the integuments and mufcles four inches long from the knee, forming a large cavity, where the bones had been fituated. This happened within lefs than five weeks from the time of the accident, during which period fomentations and poultices had been applied to the limb, and the bark given in large quantities.

The cavity in the ftump gradually filled up, and the wound was perfectly healed in feven weeks, after the feparation of the limb, forming as good a ftump as when amputation has been performed in the ufual place below the knee.

*Alcefter,*

March 15, 1787.

